# Scraps

**Published:** 1893-03

**Authors:** 


### SCRAPS

PICKED UP BY THE ASSISTANT-EDITOR.

**The Bristol Medical School.**—Mr. Augustin Prichard, as an additional note to his paper in the December number, writes as follows:

"Dr. Highett, late Mayor of Bristol, has supplied me with the following additional facts respecting medical education here before the establishment of the Bristol Medical School. He entered as a pupil at a school in College Green in 1827, where lectures on Chemistry were given by Mr. Halse, on Anatomy by Dr. Wallis, and by Dr. Davies on Medicine. This establishment was afterwards removed to the back of College Street: and it is thus that we explain the date MDCCCXXVIII on the seal of the school, which otherwise appears inconsistent with the first opening of the complete school in the Old Park in 1832; and I well remember the discussion as to what date should be placed on it, at one of the Faculty meetings of the lecturers of that time."

**A Far-seeing Specialist.**—A specialist in throat-affections was called to treat a lady, who manifested so much interest in his surgical instruments that he explained their uses to her. "This laryngoscope," said he, "is fitted with small mirrors and an electric light; the interior of your throat will be seen by me as clearly as the exterior. You would be surprised to know how far down we can see with an instrument of this kind." The operation over, the lady appeared somewhat agitated. "Poor girl!" said her sister, who was present. "It must have been very painful!" "Oh, no—not that," whispered the lady; "but just as he fixed the instrument in place, I remembered that I had a hole in the heel of my stocking!"

It was not this lady who, upon another occasion, asked the doctor what he was able to see with the instrument, and felt uncomfortable when he replied that he could see she was sitting on a cane-seat chair.

**Clinical Records.**—(1) Un juge qui a une santé de fer va dernièrement trouver son medécin. "Vous ici," s'ecria le docteur étonné, "par quel miracle?" "Ma santé commence à m'inquiéter." "Et d'où souffrez-vous? de la tête, de l'estomac, du cœur?" "Non, tout cela est en bon état. Mais j'ai parfois des insomnies . . . pendant l'audience."—*L'Union médicale.*

(2) A contemporary prints the following:—Elderly spinster to doctor (who has been asked to call between eleven and a quarter past, at a distance of many miles from his house: "I thought, Dr. Corax, that perhaps I might as well see you, as I fancy I have got a cold, and I think I had better tell you how I caught it. It was last Friday—or was it Thursday? Which was the day when it rained so in the afternoon and evening? You don't remember? I think it was Friday, because the *Weekly News* always comes on Friday evening, and I half remember that when I came in the paper was lying on the hall-table. I wanted to post a letter to my sister who lives in Dorsetshire, to tell her how very sorry I was to hear of the loss of so many of her best poultry—a fox had got at them, and made sad havoc in the poultry-yard—but that is neither here nor there—and Sarah being out, I slipped on my cloak and ran across to the post-office. But the ground was very wet after all the rain that had fallen that afternoon, and, not being able to find my goloshes——" Doctor, breaking in: "Exactly. You got your feet wet—a most common cause of a cold. I shall be able——" Patient: "Please hear me out. As I could not find my goloshes, I put on a pair of thick felt boots, which could not have let any water in, so I am sure it did not arise from my getting my feet wet. But just as I got to the post-office door a gust of wind blew my bonnet off." Doctor: "A perfectly clear statement. I will now——" Patient: "Will you allow me to explain myself? After all I do not believe that I got the chill in this way. Over and over again I have told Sarah by no means to allow the passage-window to be open on cold or wet nights. Only a few days ago I said to her: 'Sarah, you may be used to it, but I am not. In this house my will is law. I desire you to pay attention to what I have said about the passage-window.' 'Yes, ma'am,' says Sarah, as uppish as you please; but——" Doctor (who has got his thermometer ready): "Ah yes; exactly. Allow me to place my thermometer under the tongue; and will you kindly keep the mouth closed for the three minutes that are necessary for ascertaining your precise temperature. I will prescribe at once." During the three minutes the prescription is written, the doctor puts on his gloves, gathers up his hat and umbrella, and notes that there are no obstacles in the way of escape. All being prepared, he ejaculates emphatically: "Thank you. A normal temperature, I see. Good morning. You will find yourself quite well to-morrow, after taking what I have prescribed," and vanishes.

**Medical Philology** (V).—The *Catholicon* of Johannes de Janua supplied the compiler of the *Promptorium* with the following definition:

BLEDYNGE boyste. *Ventosa, guna.*

Mr. Way's note is:—

The *Catholicon* gives the following explanation: "*Guna vel guina, vas vitreum, quod et Latinis a similitudine cucurbitæ ventosa vocatur, quæ animata spiritu per igniculum in superficiem trahit sanguinem,*" PAPIAS; see Ducange. The operation of cupping, which is one of ancient use, was doubtless well known to the Friar of Lynn, who compiled the Promptorium, as one of the means resorted to when, according to the monastic institutions, there were at stated seasons *(temporibus minucionis)* general blood-lettings. See Martene de Antiq. Ritibus, and Mr. Rokewode's note on Chron. Joc. de Brakelonda, p. 11. In the Chirurgica of John Arderne, surgeon to Edw. III. where he speaks of cupping, "*ventosacio,*" a representation is given of the bledynge boyste. Sloane MS. 65, f. 70.

The quotation given by Mr. Way from Johannes Januensis shows that an early meaning of *boyste* was a glass vessel. The word—spelled in various ways, of which one was *boist*, marking its alliance with the French *boiste*—was at a later date "chiefly used of a box for ointment, a vase or flask of oil." To show this, the *New English Dictionary* cites from Barbour (1375?), *St. Nicolaus*, 294, "Scho has brocht A boyst of oyle"; and from Lonelich (*c.* 1450), *Grail*, xvii., 131, "The awngell took a boist with oynement anon." The word became used interchangeably with *ampulla*, a term generally reserved for the vessel containing holy water or oil used for purposes of sacred unction. This will be seen from the following quotation which Mr. Way gives from the *Ortus Vocabulorum* (1500), the first Latin-English Dictionary printed in this country: "*Lechitus est vas olei amplum, vel ampulla ampla que auricalco solet fieri, Anglice*, a boyste or kytte for oyle." It was also specially applied to another sacred receptacle, the pyx, in which the bread of the Holy Eucharist was preserved to be given to the sick and infirm, who could not communicate in church. Under "Boyste, or box," the *Promptorium* gives "*pix alabastrum*"; and as the equivalents of "Buyste" and "Bust," the *Catholicon Anglicum* gives "*alabastrum, alabastratum, pixis, hostiarium pro hostijs.*" Although sometimes confused with it, the pyx must be carefully distinguished from the pax, for the theft of which Bardolph (*Henry V.*, III. vi. 42) suffered capital punishment. Mr. Herrtage, in a note in the *Catholicon Anglicum*, p. 49, quotes from *English Metrical Homilies*, p. 148, where "the devil is described as passing a certain hermit's cell, and we are told that

'Boystes on himsele he bare, And ampolies als leche ware.'"

It is of interest to note that *ampulla*, literally meaning a two-handled vessel, such as was used by the Romans to contain the oil for use after bathing, being generally in shape bellied or rounded, came to signify anything puffed out, as in Horace (*Ars Poet.*, 97), "*Projicit ampullas et sesquipedalia verba.*" It is, therefore, no matter of surprise to find "Boast" defined in the *Catholicon Anglicum* as "a Boste; *ampulla, iactantia, pompa, magnificenia.*"

As we have now the verb "cup," derived from the vessel used in the operation of cupping, so in Middle English there was a verb "boyston" which came into the *Promptorium* from the *Vocabularium* written by Uguitio, Bishop of Ferrara, in the latter half of the twelfth century. The word there appears with the Latin equivalents of "*Scaro, ventoso.*"

